# Increased Glycemic Variability Evaluated by Continuous Glucose Monitoring is Associated with Osteoporosis in Type 2 Diabetic Patients

**DOI:** 10.3389/fendo.2022.861131

**Published:** 2022-06-06

**Authors:** Rong Huang, Huiying Wang, Ziyang Shen, Tingting Cai, Yunting Zhou, Yuming Wang, Wenqing Xia, Bo Ding, Rengna Yan, Huiqin Li, Jindan Wu, Jianhua Ma

**Affiliations:** Department of Endocrinology, Nanjing First Hospital, Nanjing Medical University, Nanjing, China

**Keywords:** type 2 diabetes mellitus, osteoporosis, continuous glucose monitoring, glycemic variability, mean amplitude of glycemic excursions

## Abstract

**Background:**

Subjects with type 2 diabetes mellitus (T2DM) are susceptible to osteoporosis. This study was conducted to evaluate the association between glycemic variability evaluated by continuous glucose monitoring (CGM) and osteoporosis in type 2 diabetic patient.

**Methods:**

A total of 362 type 2 diabetic subjects who underwent bone mineral density (BMD) measurement and were monitored by a CGM system from Jan 2019 to May 2020 were enrolled in this cross-sectional study. Glycemic variability was calculated with the Easy GV software, including 24-hour mean blood glucose (24-h MBG), the standard deviation of 24-h MBG (SDBG), coefficient of variation (CV), mean amplitude of glycemic excursions (MAGE), and time in range between 3.9 and 10.0 mmol/L (TIR). Other potential influence factors for osteoporosis were also examined.

**Results:**

Based on the T-scores of BMD measurement, there were 190 patients with normal bone mass, 132 patients with osteopenia and 40 patients with osteoporosis. T2DM patients with osteoporosis showed a higher 24-h MBG, SDBG, CV, and MAGE, but a lower TIR (all *p* < 0.05). Multivariate logistic regression analysis revealed that age, female gender, body mass index (BMI), low-density lipoprotein cholesterol (LDL-C), serum uric acid (SUA) and MAGE independently contribute to osteoporosis, and corresponding odds ratio [95% confidence interval (CI)] was 1.129 (1.072-1.190), 4.215 (1.613-11.012), 0.801 (0.712-0.901), 2.743 (1.385-5.431), 0.993 (0.988-0.999), and 1.380 (1.026-1.857), respectively. Further receiver operating characteristic analysis with Youden index indicated that the area under the curve and its 95% CI were 0.673 and 0.604-0.742, with the optimal cut-off value of MAGE predicting osteoporosis being 4.31 mmol/L.

**Conclusion:**

In addition to conventional influence factors including age, female gender, BMI, LDL-C and SUA, increased glycemic variability assessed by MAGE is associated with osteoporosis in type 2 diabetic patients.

## Introduction

Both type 2 diabetes mellitus (T2DM) and osteoporosis are frequent metabolic disorders, and induces high morbidity and mortality in elderly population (1, 2). It has become apparent that subjects with T2DM are susceptible to osteoporosis (3). A meta-analysis of 54 studies also showed more than one-third of T2DM patients suffered from osteoporosis in Chinese mainland (4). Therefore, great efforts have been exerted to explore potential risk factors for osteoporosis in patients with T2DM, including increasing age, female gender, unhealthy lifestyle, insulin resistance, elevated body mass index (BMI) and glycated hemoglobin (HbA1c) (5–7). However, exact risk factors and mechanisms remain unknown.

HbA1c, the “gold standard” for long-term glycemic control for decades, has been demonstrated to be positively associated with diabetic chronic complications. A cross-sectional study by Sun et al. strengthened that diabetes duration and HbA1c are risk factors for diabetic retinopathy (DR) (8). Results from Atkin et al. study showed that the HbA1c threshold of 6.5% for DR also elevated the risk of diabetic nephropathy (DN) (9). The INTERPRET-DD study involved in 2,733 subjects with T2DM from 14 countries revealed that each 1% increase in HbA1c level generates an 11 % increased risk of diabetic peripheral neuropathy (DPN) (10). Similarly, poor glycemic control evaluated by HbA1c was linked to lower concentrations of bone formation biomarkers, increasing the susceptibility to osteoporosis in type 2 diabetic postmenopausal women (11, 12). Xu et al. demonstrated that HbA1c is correlated with increased risk of osteoporosis, as well as osteopenia (6). In the study by Wen et al., higher HbA1c level could increase the risk of osteoporosis in patients with newly diagnosed T2DM, which indicated that bone metabolism has been damaged by elevated glucose level even in the early stage of T2DM (7).

During recent years, glycemic variability gained much research interests, which reflected more comprehensive glycemic control. Apart from conventional risk factors including diabetic duration, HbA1c and homeostatic model assessment of insulin resistance (HOMA-IR), Hu et al. found that increased glycemic variability assessed by mean amplitude of glycemic excursions (MAGE) independently contribute to DPN in patients with T2DM (13). Even in T2DM patients with well-controlled HbA1c, MAGE was also demonstrated to be a significantly independent contributor to DPN (14). In Sartore et al. study, glycemic variability regardless of HbA1c may be associated with the development of DR, particularly in the case of acute fluctuations and acute hyperglycemia (15). However, few studies have been performed to assess the relationship of glycemic variability with osteoporosis. Therefore, we conducted this study to evaluate the association between glycemic variability evaluated by continuous glucose monitoring (CGM) system and the risk of osteoporosis in patients with T2DM aged ≥50 years.

## Methods

### Study Population

This cross-sectional observational study was conducted at Department of Endocrinology, Nanjing First Hospital from Jan 2019 to May 2020. The study was approved by the Research Ethics Committee of Nanjing First Hospital in accordance with the Declaration of Helsinki (KY20170904-07), and all participants signed written informed consent. The patients who met the following criteria were included (1): diagnosis of T2DM according to the World Health Organization (1999) criteria (2); ≥ 50 years old; and (3) subjects who underwent bone mineral density (BMD) measurement and CGM. The exclusion criteria included the following: (1) patients with diabetic acute complications (i.e., diabetic ketoacidosis, diabetic hyperosmolar coma, or severe hypoglycemic events); (2) patients with severe liver diseases (a history of liver dysfunction or liver enzyme level more than two times the upper limit of normal), or kidney diseases (a history of renal dysfunction or the estimated glomerular filtration rate (eGFR) less than 60 ml/min/1.73m^2^); (3) patients with hyperparathyroidism, rheumatoid arthritis, or other endocrine and immune diseases; (4) patients with malignant tumors or mental disorders; (5) patients using medications that might influence bone metabolism (i.e., bisphosphonates, estrogens, selective estrogen receptor modulators (SERMs), calcitonin, calcium, vitamin D, glucocorticoid or immunosuppressive agents); and (6) no sufficient data to calculate glycemic variability.

Our sample size was calculated using PASS version 21.0.3 software (NCSS, LLC, Kaysville, Utah, USA). If we set type I error (α) as 0.05 and permissible error (δ) as 5% (assuming 20% data missing), the sample size should be at least 293 based on the prevalence of osteoporosis derived from a recent Chinese cross-sectional study (3). In this study, a total of 362 type 2 diabetic patients with complete information were ultimately enrolled.

### Clinical Measurement

A questionnaire was employed to collect the information about age, gender, diabetes duration, lifestyle behaviors (i.e., smoking and drinking), and medication usage (i.e., insulin, oral hypoglycemic agents (OHA) and both). All participants underwent anthropometric measurements including height, weight, waist circumstance, hip circumstance and blood pressure. BMI was calculated as weight divided by height squared (kg/m^2^), and waist-to-hip ratio (WHR) was calculated as the ratio of waist circumstance to hip circumstance. Hypertension was defined as systolic blood pressure (SBP) ≥ 140 mmHg and/or diastolic blood pressure (DBP) ≥ 90 mmHg and/or a diagnosis of hypertension currently taking antihypertensive agents. Fasting blood samples were obtained for measuring fasting blood glucose (FBG), HbA1c, triglycerides (TG), total cholesterol (TC), high-density lipoprotein cholesterol (HDL-C), low-density lipoprotein cholesterol (LDL-C), serum uric acid (SUA) and serum creatinine (SCr). The eGFR was determined based on the Modification of Diet in Renal Disease (MDRD) Study formula: eGFR = 175 × (Scr) ^–1.154^ × (age) ^–0.203^ (× 0.742 if female) (16).

### CGM Measurement

All eligible subjects had CGM (Medtronic Incorporated, Northridge, Minnesota, USA) monitored for a continuous 72h. The sensor of the CGM system monitor was inserted into anterior abdominal skin, and calibrated by at least four finger-tip capillary blood glucose readings per day. Data obtained from 0:00 to 24:00 of the second day were calculated for glycemic variability with the Easy GV software, including 24-hour mean blood glucose (24-h MBG), the standard deviation of 24-h MBG (SDBG), coefficient of variation (CV), MAGE, and time in range between 3.9 and 10.0 mmol/L (TIR).

### BMD Measurement

The BMD values along with T-scores and Z-scores at the lumbar spine, femur neck, and total hip were measured using dual-energy X-ray absorptiometry (DXA) (GE lunar prodigy, GE, Madison, MA, USA; coefficient of variation = 0.30%). The T-scores were calculated with the gender-matched BMD data from healthy Asian young adults provided by the DXA equipment manufacturer. Osteoporosis was diagnosed based on the World Health Organization (WHO) T-score criteria (17). Briefly, a T-score ≥ −1.0 indicated normal, osteopenia was defined by -2.5 < T-score < −1.0, and osteoporosis was diagnosed as a T-score ≤ −2.5.

### Statistical Analysis

Data were presented as mean ± standard deviation, median (interquartile range), or n (%) as appropriate. Analysis of variance (ANOVA) was applied in normally distributed data, whereas Kruskal-Wallis H test was used in asymmetrically distributed data. The frequencies of categorical variables were compared with Pearson’s chi-squared (χ^2^) test. *Post hoc* analyses were further carried out for pairwise comparisons if there were between-group differences. Univariate logistic regression analysis was used to investigate potential indicators affecting osteoporosis. Multivariable logistic regression analysis was performed to determine independent factors associated with osteoporosis susceptibility. Correlation analyses were also performed between BMD value and clinical characteristics and CGM parameters of the enrolled subjects, then stepwise multiple linear regression analyses were also run with BMD value as the dependent variable. Receiver operating characteristic (ROC) curve was employed to present the area under the curve (AUC) with corresponding 95% CI, and the Youden index was calculated to identify the optimal cut-off point of involved glycemic variability parameters for predicting diabetic osteoporosis, as well as its sensitivity and specificity. Statistical analyses were performed with SPSS version 22.0 (SPSS Inc., Chicago, IL, USA), and a *p*-value < 0.05 was considered statistically significant.

## Results

### Clinical Characteristics and CGM Parameters of the Enrolled Subjects

**Table 1** summarizes the clinical characteristics and CGM parameters of the enrolled T2DM subjects. Based on the T-scores of BMD measurement, there are 190 patients with normal bone mass, 132 patients with osteopenia and 40 patients with osteoporosis. T2DM patients with osteoporosis were older, and had higher ratio of female gender, HbA1c, TC, HDL-C and LDL-C levels, but lower BMI and SUA levels (all *p* < 0.05). Regarding the parameters of glycemic variability, T2DM patients with osteoporosis showed higher 24-h MBG, SDBG, CV, and MAGE, but a lower TIR (all *p* < 0.05). *Post hoc* analyses revealed that there exist significant differences for all above-mentioned variables between normal and osteoporosis subjects, while for age, gender, and BMI between normal and osteopenia subjects, and gender, BMI, TC, HDL-C, LDL-C, SUA, 24-h MBG, and MAGE between osteopenia and osteoporosis subjects (all *p* < 0.05 after Bonferroni correction). Ratio of smoking and drinking, WHR, SBP, DBP, FBG, eGFR and usage of OHA, insulin or both were comparable among the three groups (all *p* > 0.05).

**Table 1:** Clinical characteristics and CGM parameters of the enrolled subjects.

	Normal (n = 190)	Osteopenia (n = 132)	Osteoporosis (n = 40)	*p* value
Age (years)	61.00 (56.00-66.25)	64.00 (58.00-70.75)	69.50 (58.00-77.00)	<0.001^a,b^
Female, n (%)	77 (40.53)	72 (54.55)	32 (80.00)	<0.001^a,b,c^
Smoking, n (%)	49 (25.79)	31 (23.48)	5 (12.50)	0.197
Drinking, n (%)	23 (12.11)	15 (11.36)	2 (5.00)	0.424
BMI (kg/m^2^)	24.35 (22.84-26.73)	23.57 (21.15-25.37)	21.49 (18.76-24.60)	<0.001^a,b,c^
WHR	0.94 (0.91-0.96)	0.94 (0.91-0.96)	0.92 (0.88-0.95)	0.067
SBP (mmHg)	130.00 (120.00-140.00)	130.00 (120.00-140.00)	130.00 (120.00-140.00)	0.731
DBP (mmHg)	80.00 (70.00-84.00)	80.00 (70.00-80.00)	80.00 (70.00-80.00)	0.619
Diabetes duration (years)	9.00 (5.00-12.00)	10.00 (5.00-12.75)	10.00 (7.25-15.00)	0.112
FBG (mmol/L)	7.19 (6.14-9.36)	7.69 (6.23-9.82)	7.09 (6.13-11.40)	0.506
HbA1c (%)	7.80 (7.00-9.23)	8.25 (7.30-9.28)	9.05 (7.65-10.20)	0.010^b^
TG (mmol/L)	1.35 (0.90-1.84)	1.25 (0.86-1.69)	1.00 (0.79-1.68)	0.154
TC (mmol/L)	4.15 (3.33-5.01)	4.11 (3.51-5.17)	4.73 (4.16-5.76)	0.003^b,c^
HDL-C (mmol/L)	1.19 (1.01-1.41)	1.17 (1.02-1.40)	1.38 (1.08-1.63)	0.026^b,c^
LDL-C (mmol/L)	1.85 ± 0.65	1.84 ± 0.61	2.25 ± 0.58	0.001^b,c^
SUA (μmol/L)	291.00 (234.00-354.00)	274.00 (215.75-327.50)	228.50 (173.00-270.25)	<0.001^b,c^
eGFR [mL/(min·1.73 m^2^)]	95.68 (82.89-111.46)	100.03 (85.19-113.71)	108.65 (85.08-122.59)	0.108
Use antidiabetic agents, n (%)	189 (99.47)	131 (99.24)	40 (100.00)	0.850
OHA	115 (60.85)	66 (50.38)	20 (50.00)	0.132
Insulin	24 (12.70)	26 (19.85)	4 (10.00)	0.136
Both	50 (26.45)	39 (29.77)	16 (40.00)	0.227
CGM parameters				
24-h MBG (mmol/L)	8.78 (7.71-11.03)	9.04 (7.79-10.79)	10.14 (8.70-11.51)	0.019^b,c^
SDBG (mmol/L)	1.81 (1.34-2.48)	2.03 (1.51-2.79)	2.36 (1.85-3.09)	<0.001^b^
CV (%)	20.25 (15.53-26.11)	22.04 (17.39-28.25)	23.85 (20.45-29.94)	0.003^b^
MAGE (mmol/L)	4.39 (3.32-6.13)	4.83 (3.70-6.97)	5.83 (4.72-7.03)	<0.001^b,c^
TIR (%)	75.87 (37.59-90.19)	65.10 (39.41-88.54)	53.30 (26.39-73.96)	0.010^b^

Data are presented as mean ± standard deviation, median (interquartile range) or n (%) as appropriate. Analysis of variance (ANOVA) for comparison of normally distributed data; Kruskal-Wallis H test for comparison of asymmetrically distributed data; Pearson’s chi-squared (χ^2^) test for comparison of categorical variables. ^a^, p < 0.05 for post hoc analysis between Normal and Osteopenia groups; ^b^, p < 0.05 for post hoc analysis between Normal and Osteoporosis groups; ^c^, p < 0.05 for post hoc analysis between Osteopenia and Osteoporosis groups. CGM, continuous glucose monitoring; BMI, body mass index; WHR, waist-to-hip ratio; SBP, systolic blood pressure; DBP, diastolic blood pressure; FBG, fasting blood glucose; HbA1c, glycosylated hemoglobin; TG, triglyceride; TC, total cholesterol; HDL-C, high-density lipoprotein cholesterol; LDL-C, low-density lipoprotein cholesterol; SUA, serum uric acid; eGFR, estimated glomerular filtration rate; OHA, oral hypoglycemic agents; 24-h MBG, 24-hour mean blood glucose; SDBG, the standard deviation of 24-h MBG; CV, coefficient of variation; MAGE, mean amplitude of glycemic excursion; TIR, time in range between 3.9 and 10.0 mmol/L.

### Association of Glycemic Variability and Clinical Characteristics With Osteoporosis in T2DM Patients

Univariate logistic regression analysis was performed to explore potential factors for osteoporosis in T2DM patients. As shown in **Table 2**, osteoporosis had a positive association with age [odds ratios (95% confidence interval) (OR (95% CI)), 1.083 (1.041-1.127)], female gender [5.870 (2.567-13.423)], diabetes duration [1.063 (1.009-1.120)], HbA1c [1.401 (1.175-1.671)], HDL-C [3.861 (1.524-9.786)], LDL-C [2.647 (1.526-4.590)], 24-h MBG [1.206 (1.045-1.392)], SDBG [1.988 (1.378-2.868)], CV [1.062 (1.020-1.106)], and MAGE [1.306 (1.129-1.511)]. However, BMI [0.799 (0.719-0.889)], SUA [0.988 (0.984-0.993)], and TIR [0.995 (0.991-0.998)] were negatively associated with osteoporosis in T2DM subjects.

**Table 2:** Univariate logistic regression analysis to explore potential factors for osteoporosis in T2DM patients.

	OR (95% CI)	*p* value
Age	1.083 (1.041-1.127)	<0.001
Female	5.870 (2.567-13.423)	<0.001
BMI	0.799 (0.719-0.889)	<0.001
WHR	0.069 (0.001-3.633)	0.186
SBP	0.993 (0.972-1.015)	0.517
DBP	0.983 (0.949-1.019)	0.359
Diabetes duration	1.063 (1.009-1.120)	0.021
FBG	1.084 (0.970-1.211)	0.155
HbA1c	1.401 (1.175-1.671)	<0.001
TG	0.745 (0.488-1.138)	0.173
TC	1.161 (0.963-1.401)	0.118
HDL-C	3.861 (1.524-9.786)	0.004
LDL-C	2.647 (1.526-4.590)	0.001
SUA	0.988 (0.984-0.993)	<0.001
eGFR	1.013 (0.999-1.028)	0.070
24-h MBG	1.206 (1.045-1.392)	0.010
SDBG	1.988 (1.378-2.868)	<0.001
CV	1.062 (1.020-1.106)	0.004
MAGE	1.306 (1.129-1.511)	<0.001
TIR	0.995 (0.991-0.998)	0.006

T2DM, type 2 diabetes mellitus; OR, odds ratio; CI, confidence interval; BMI, body mass index; WHR, waist-to-hip ratio; SBP, systolic blood pressure; DBP, diastolic blood pressure; FBG, fasting blood glucose; HbA1c, glycosylated hemoglobin; TG, triglyceride; TC, total cholesterol; HDL-C, high-density lipoprotein cholesterol; LDL-C, low-density lipoprotein cholesterol; SUA, serum uric acid; eGFR, estimated glomerular filtration rate; OHA, oral hypoglycemic agents; 24-h MBG, 24-hour mean blood glucose; SDBG, the standard deviation of 24-h MBG; CV, coefficient of variation; MAGE, mean amplitude of glycemic excursion; TIR, time in range between 3.9 and 10.0 mmol/L.

### Multivariate Logistic Regression Analysis to Determine Independent Factors Associated With Osteoporosis Susceptibility in T2DM Patients

Osteoporosis was significantly correlated with age, female gender, BMI, diabetes duration, HbA1c, HDL-C, LDL-C, SUA, 24-h MBG, SDBG, CV, MAGE and TIR according to the above-mentioned univariate logistic regression analysis. Then, a multivariate logistic regression analysis was carried out to determine independent factors associated with osteoporosis susceptibility in T2DM patients. Results demonstrated that age, female gender, BMI, LDL-C, SUA and MAGE independently contribute to osteoporosis, with corresponding OR (95% CI) being 1.129 (1.072-1.190), 4.215 (1.613-11.012), 0.801 (0.712-0.901), 2.743 (1.385-5.431), 0.993 (0.988-0.999), and 1.380 (1.026-1.857), respectively (**Table 3**). Due to the relatively small sample size of subjects with osteoporosis, stepwise multiple linear regression analyses were also run with BMD value as the dependent variable, and the results were in substantial concordance with our findings with osteoporosis as outcome (**Tables S1**, **S2**).

**Table 3:** Multivariate logistic regression analysis to determine independent factors associated with osteoporosis susceptibility in T2DM patients.

	B	SE	Wald	OR (95% CI)	*p* value
Age	0.121	0.027	20.756	1.129 (1.072-1.190)	<0.001
Female	1.439	0.490	8.619	4.215 (1.613-11.012)	0.003
BMI	-0.222	0.060	13.635	0.801 (0.712-0.901)	<0.001
LDL-C	1.009	0.349	8.380	2.743 (1.385-5.431)	0.004
SUA	-0.007	0.003	5.161	0.993 (0.988-0.999)	0.023
MAGE	0.322	0.151	4.541	1.380 (1.026-1.857)	0.033

OR, odds ratios; CI, confidence interval; BMI, body mass index; LDL-C, low-density lipoprotein cholesterol; SUA, serum uric acid; MAGE, mean amplitude of glycemic excursion.

### ROC Analysis to Identify the Optimal Cut-Off Value of MAGE Predicting Osteoporosis in T2DM Patients

**Figure 1:**
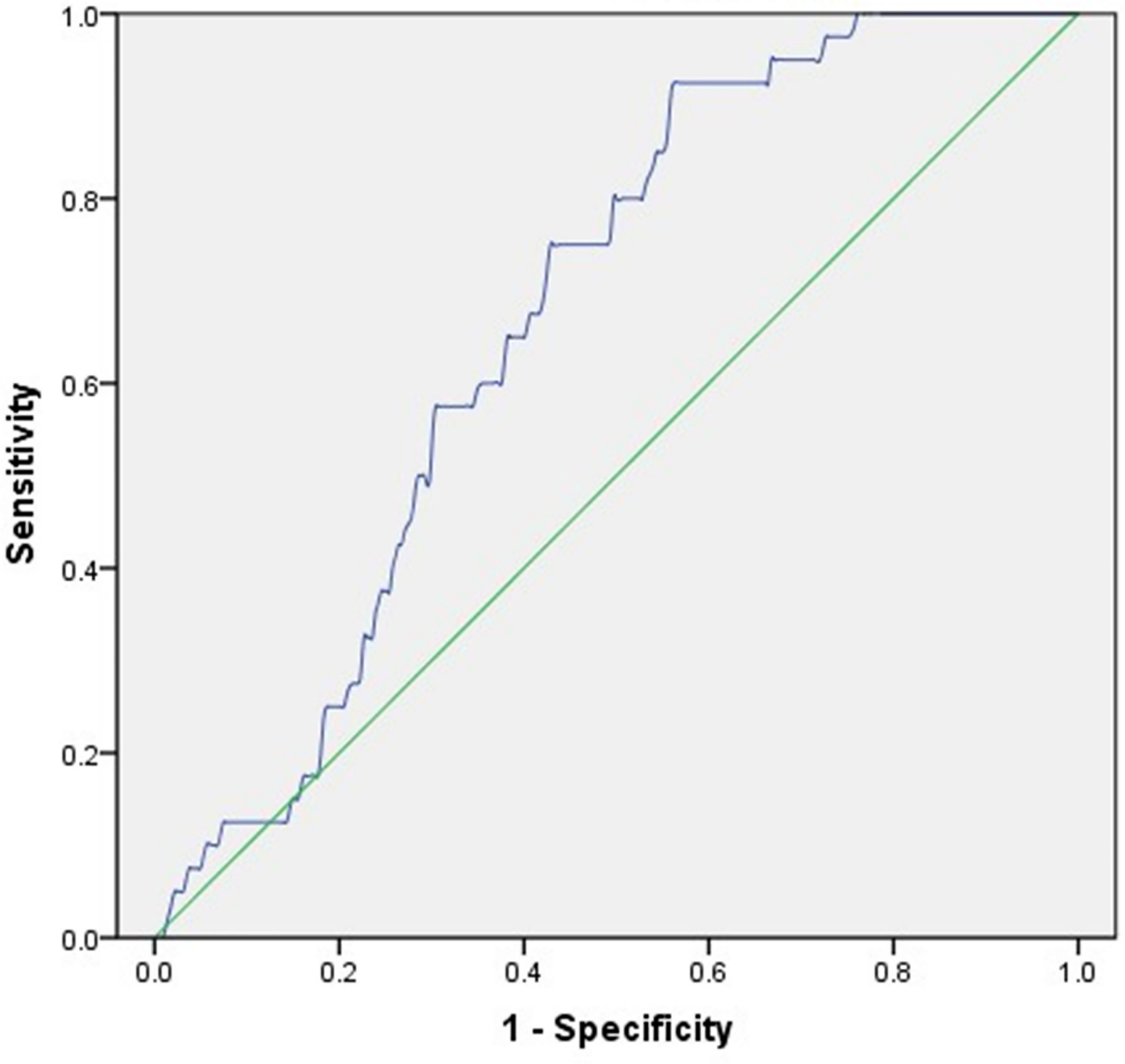
ROC analysis employed to identify the optimal cut-off value of MAGE to predict osteoporosis in T2DM patients. AUC (95% CI) = 0.673 (0.604-0.742), cut-off point = 4.31 mmol/L, Youden index = 0.366, sensitivity = 92.50%, specificity = 55.90%.

Regardless of HbA1c, increased glycemic variability assessed by MAGE was associated with osteoporosis. Then, ROC analysis and the Youden index were employed to identify the optimal cut-off value of MAGE to predict osteoporosis in T2DM patients. The corresponding AUC and its 95% CI were 0.673 and 0.604-0.742, with the optimal MAGE cut-off value predicting osteoporosis being 4.31 mmol/L (**Figure 1**).

## Discussion

To our certain knowledge, this study is the first to reveal the relationship between CGM-assessed glycemic variability and the susceptibility to osteoporosis in type 2 diabetic patients. Among a population of 362 subjects with T2DM, we observed 24-h MBG, SDBG, CV, and MAGE were positively associated with osteoporosis, while TIR was inversely related to osteoporosis. Further multivariate logistic regression analysis revealed that MAGE was an independent contributor to osteoporosis regardless of HbA1c, with each 1-mmol/L increment of MAGE being associated with a 38% higher relative risk of osteoporosis. ROC analysis demonstrated that the optimal MAGE cut-off value predicting osteoporosis was 4.31 mmol/L. In addition, age, female gender and LDL-C were also independent risk factors, while BMI and SUA were independent protective factors for osteoporosis in type 2 diabetic patients.

As HbA1c reflects average glucose levels over the past 2-3 months, glycemic variability as assessed by CGM system may reflect more accurately glucose profiles. Importantly, MAGE was first demonstrated to be an independent contributor to osteoporosis in patients with T2DM in the current study. The exact mechanisms remain unknown. One possible reason was that higher MAGE may reflect increased ratio of acute hyperglycemia, while acute hyperglycemia was capable of inducing alkaline phosphatase and suppressing osteocalcin in differentiated osteoblasts (18). Second, increased glycemic variability was also linked to lower BMI in newly diagnosed Chinese type 2 diabetic patients, and low BMI was proved to be a risk factor for osteoporosis (19). Third, results derived from a cross-sectional study showed that osteoporotic women had markedly lower antioxidant levels (20). MAGE has also been linked to high-sensitivity C-reactive protein (hs-CRP) and diacron-reactive oxygen metabolites (d-ROMs), which raise the possibility that MAGE may contribute to bone loss and osteoporosis *via* promoting oxidative stress and inflammatory status (21, 22). Additionally, studies have shown potential effect of gut microbiota on BMD and osteoporosis, and plasma glucose level was identified to contribute significantly to a differentiated gut microbiota structure (23–25). Therefore, gut microbiota may mediate osteoporosis induced by MAGE.

In this study, we also reported that age, female gender, and BMI are independently associated with osteoporosis in patients with T2DM. The results were partially consistent with a previous study conducted in healthy volunteers, which confirmed that age, sex, and BMI are significant predictors of osteoporosis in Chinese (26). Postmenopausal women have been generally recognized as the population with high risk of osteoporosis, which may be mainly explained by an obvious decline in estrogen level (27). Most previous studies also proposed a high BMI to exert a protective factor for osteoporosis (28–30). One possible reason was that adipocytes are important for estrogen production sources, and a higher BMI may indirectly affect osteoblast and osteoclast activity by producing more estrogen sources (31). Additionally, a higher BMI partly reflects more subcutaneous fat, which exerts beneficial roles in bone structure and strength (32).

The association between serum lipids and bone metabolism has been extensively investigated but shown conflicting findings. Our study found that higher LDL-C level is independently associated with osteoporosis in type 2 diabetic patients. Cui et al. and Li et al. both revealed that elevated serum HDL-C level has a greater probability of being osteoporosis, but no correlations between LDL-C, TC, and TG and osteoporosis (26–33). In a cross-sectional study by Zhang et al., non-linear relationships were found of TC, LDL-C, HDL-C with lumbar spine BMD in postmenopausal women (34). Results from an epidemiological study in South Korea pre-menopausal and post-menopausal women exhibited a negative correlation between serum TC, LDL-C levels and BMD (35). A Mendelian randomization study also observed causal effect of LDL-C to BMD (36). Possibly, different sources and number of participants, inclusion of confounding factors, and methods of analysis contributed to the inconsistent results. Increased lipid levels may bring about progressive oxidation accumulation in the subendothelial matrix of bone vessels, while these oxidized lipids inhibited the differentiation and mineralization of bone cells (37). Statins, a class of drugs that primarily lower LDL, could particularly intervene in bone turnover and remodeling *via* acting on bone marrow mesenchymal stem cells and osteoclasts (38).

Consistent with our study, positive association was found between SUA and BMD, which suggested SUA is protective against osteoporosis in type 2 diabetic patients (39–42). A 6-year longitudinal study also demonstrated that lower SUA level is associated with higher occurrence of at least osteopenia in Chinese type 2 diabetic patients (43). In subjects without T2DM, SUA was also revealed to be positively correlated with BMD (44–46). Whereas in subgroup analyses according to ethnicity by Yao et al, an inverted U-shaped curve relationship was found about the association of SUA with lumbar BMD in blacks (47). *In vitro* study has shown promotion of proliferation and osteogenic differentiation in human mesenchymal stem cells with the increased concentration of SUA (48). As reduced BMD is related to increased level of oxidative stress and inflammation, SUA might also protect against osteoporosis *via* antioxidant capacity (49, 50). However, SUA is a metabolic waste product of purine, and hyperuricemia is a main cause of gout, which supported the phenomenon that subjects with SUA over than 7.5mg/dL is susceptible to osteoporosis (51).

However, several limitations should be noted in the current study. First, we could not draw the conclusion whether MAGE is a cause or an effect for osteoporosis in type 2 diabetic patients as this was a cross-sectional study. In addition, the 3-day CGM-based MAGE may not fully represent glycemic control of the included subjects in peacetime. Second, only hospital-based Chinese Han population were enrolled in the present study, therefore, our results may not be generalizable to all patients from other ethnic groups. Third, only 40 T2DM subjects with osteoporosis, especially among women and with a rather large number of confounders were included in our study, which may limit the power of the study. Although consistent results were drawn with BMD value as the dependent variable by stepwise multiple linear regression analyses, further studies with a substantial sample size should be divided into a training set and a validation set, as well as a validation in a heterogeneous population using Machine Learning algorithms is needed to verify this finding. Then, serum estrogen level as well as some bone turnover markers were not measured in this study, including procollagen type I N-terminal propeptide (P1NP), β-Cross Laps of type I collagen containing cross-linked C-telopeptide (β-CTX) and osteocalcin. Moreover, physical activity and dietary patterns, which could potentially affect glycemic variability, were not evaluated in our study.

In conclusion, increased glycemic variability assessed by MAGE is associated with osteoporosis in patients with T2DM, in addition to conventional influence factors including age, female gender, BMI, LDL-C and SUA. Further well-designed prospective cohort studies are warranted to confirm this observed association, and to determine the causality of MAGE with the onset and progression of osteoporosis in type 2 diabetic patients.

## Data Availability Statement

The original contributions presented in the study are included in the article/**Supplementary Material**. Further inquiries can be directed to the corresponding author.

## Ethics Statement

The studies involving human participants were reviewed and approved by the Research Ethics Committee of Nanjing First Hospital, Nanjing Medical University. The patients/participants provided their written informed consent to participate in this study.

## Author Contributions

JM and RH designed the study. RH, HW, ZS, TC, YZ and YW collected the data, RH, HW and ZS performed the analyses. RH, WX, BD and RY wrote the first draft. And HL, JW and JM checked the manuscript and revised it. All authors approved the final submission.

## Funding

This work was supported by the National Key R&D Program of China (No. 2018YFC1314100), the National Natural Science Foundation of China (No. 81870563) and the Science and Technology Development Fund of Nanjing Medical University (No. NMUB2020209).

## Conflict of Interest

The authors declare that the research was conducted in the absence of any commercial or financial relationships that could be construed as a potential conflict of interest.

## Publisher’s Note

All claims expressed in this article are solely those of the authors and do not necessarily represent those of their affiliated organizations, or those of the publisher, the editors and the reviewers. Any product that may be evaluated in this article, or claim that may be made by its manufacturer, is not guaranteed or endorsed by the publisher.
