# Supplementary material for: Increased Glycemic Variability Evaluated by Continuous Glucose Monitoring is Associated with Osteoporosis in Type 2 Diabetic Patients
Source: Front Endocrinol (Lausanne). 2022 Jun 6;13:861131. doi: 10.3389/fendo.2022.861131 (PMC9207512; doi:10.3389/fendo.2022.861131)
Supplement: Supplementary file 1 [file Table_1.docx]

Table S1 Relationship of BMD value with clinical characteristics and CGM parameters of the enrolled subjects

|  | lumbar spine | |  | femur neck | |  | total hip | |
| --- | --- | --- | --- | --- | --- | --- | --- | --- |
|  | r | *p* |  | r | *p* |  | r | *p* |
| Age | -0.104 | 0.048 |  | -0.282 | <0.001 |  | -0.246 | <0.001 |
| BMI | 0.213 | <0.001 |  | 0.201 | <0.001 |  | 0.276 | <0.001 |
| WHR | 0.151 | 0.004 |  | 0.109 | 0.037 |  | 0.165 | 0.002 |
| SBP | 0.061 | 0.247 |  | 0.054 | 0.304 |  | 0.048 | 0.364 |
| DBP | 0.020 | 0.706 |  | 0.070 | 0.183 |  | 0.063 | 0.234 |
| Diabetes duration | -0.093 | 0.077 |  | -0.105 | 0.046 |  | -0.117 | 0.026 |
| FBG | -0.067 | 0.206 |  | -0.089 | 0.090 |  | -0.085 | 0.106 |
| HbA1c | -0.113 | 0.031 |  | -0.117 | 0.025 |  | -0.165 | 0.002 |
| TG | 0.063 | 0.232 |  | 0.029 | 0.587 |  | 0.048 | 0.358 |
| TC | -0.197 | <0.001 |  | -0.154 | 0.003 |  | -0.193 | <0.001 |
| HDL-C | -0.164 | 0.002 |  | -0.050 | 0.346 |  | -0.079 | 0.135 |
| LDL-C | -0.225 | <0.001 |  | -0.164 | 0.002 ^a^ |  | -0.205 | <0.001 ^a^ |
| SUA | 0.274 | <0.001 |  | 0.224 | <0.001 |  | 0.250 | <0.001 |
| eGFR | -0.166 | 0.001 |  | -0.074 | 0.159 |  | -0.089 | 0.090 |
| 24-h MBG | -0.109 | 0.039 |  | -0.122 | 0.020 |  | -0.150 | 0.004 |
| SDBG | -0.159 | 0.002 |  | -0.183 | <0.001 |  | -0.208 | <0.001 |
| CV | -0.116 | 0.027 |  | -0.137 | 0.009 |  | -0.153 | 0.003 |
| MAGE | -0.167 | 0.036 |  | -0.185 | <0.001 |  | -0.207 | <0.001 |
| TIR | 0.110 | 0.001 |  | 0.155 | 0.003 |  | 0.185 | <0.001 |

^a^ Pearson correlation; the others, Spearman correlation.

Abbreviations: BMD, bone mineral density; CGM, continuous glucose monitoring; BMI, body mass index; WHR, waist-to-hip ratio; SBP, systolic blood pressure; DBP, diastolic blood pressure; FBG, fasting blood glucose; HbA1c, glycosylated hemoglobin; TG, triglyceride; TC, total cholesterol; HDL-C, high-density lipoprotein cholesterol; LDL-C, low-density lipoprotein cholesterol; SUA, serum uric acid; eGFR, estimated glomerular filtration rate; 24-h MBG, 24-hour mean blood glucose; SDBG, the standard deviation of 24-h MBG; CV, coefficient of variation; MAGE, mean amplitude of glycemic excursion; TIR, time in range between 3.9 and 10.0 mmol/L.
