# Supplementary material for: Increased Glycemic Variability Evaluated by Continuous Glucose Monitoring is Associated with Osteoporosis in Type 2 Diabetic Patients
Source: Front Endocrinol (Lausanne). 2022 Jun 6;13:861131. doi: 10.3389/fendo.2022.861131 (PMC9207512; doi:10.3389/fendo.2022.861131)
Supplement: Supplementary file 2 [file Table_2.docx]

Table S2 Stepwise multiple linear regression analysis with BMD value as the dependent variable

|  | Adjusted R^2^ | Unstandardized β | β Std. error | Standardized β | t | *p* value | 95% CI |
| --- | --- | --- | --- | --- | --- | --- | --- |
| Model 1 | 0.267 |  |  |  |  |  |  |
| Constant |  | 0.957 | 0.278 |  | 3.443 | 0.001 | 0.410-1.503 |
| Age |  | -0.003 | 0.001 | -0.132 | -2.723 | 0.007 | -0.005--0.001 |
| Female |  | -0.126 | 0.019 | -0.327 | -6.745 | <0.001 | -0.163--0.089 |
| BMI |  | 0.011 | 0.003 | 0.190 | 3.852 | 0.001 | 0.005-0.017 |
| LDL-C |  | -0.066 | 0.015 | -0.217 | -4.409 | <0.001 | -0.095--0.036 |
| SUA |  | 0.000 | 0.000 | 0.134 | 2.596 | 0.010 | 0.001-0.001 |
| Model 2 | 0.218 |  |  |  |  |  |  |
| Constant |  | 1.209 | 0.210 |  | 5.768 | <0.001 | 0.796-1.621 |
| Age |  | -0.005 | 0.001 | -0.295 | -5.919 | <0.001 | -0.007--0.003 |
| Female |  | -0.062 | 0.015 | -0.209 | -4.188 | <0.001 | -0.091--0.033 |
| BMI |  | 0.007 | 0.002 | 0.169 | 3.424 | 0.001 | 0.003-0.012 |
| LDL-C |  | -0.029 | 0.012 | -0.126 | -2.520 | 0.012 | -0.052--0.006 |
| MAGE |  | -0.011 | 0.005 | -0.166 | -2.126 | 0.034 | -0.021--0.001 |
| Model 3 | 0.286 |  |  |  |  |  |  |
| Constant |  | 1.021 | 0.217 |  | 4.710 | <0.001 | 0.595-1.448 |
| Age |  | -0.005 | 0.001 | -0.267 | -5.602 | <0.001 | -0.007--0.003 |
| Female |  | -0.069 | 0.015 | -0.218 | -4.556 | <0.001 | -0.099--0.039 |
| BMI |  | 0.012 | 0.002 | 0.251 | 5.324 | <0.001 | 0.008-0.016 |
| LDL-C |  | -0.040 | 0.012 | -0.161 | -3.355 | 0.001 | -0.064--0.017 |
| MAGE |  | -0.013 | 0.005 | -0.189 | -2.529 | 0.012 | -0.024--0.003 |

Model 1, Stepwise multiple linear regression analysis with BMD value of the lumbar spine as the dependent variable; Model 2, Stepwise multiple linear regression analysis with BMD value of the femur neck as the dependent variable; Model 3, Stepwise multiple linear regression analysis with BMD value of the total hip as the dependent variable.

Abbreviations: BMD, bone mineral density; CI, confidence interval; BMI, body mass index; LDL-C, low-density lipoprotein cholesterol; SUA, serum uric acid; MAGE, mean amplitude of glycemic excursion.
